# The Role of Interleukin 18/Interleukin 18-Binding Protein in Adult-Onset Still’s Disease and Systemic Juvenile Idiopathic Arthritis

**DOI:** 10.3390/jcm11020430

**Published:** 2022-01-15

**Authors:** Charlotte Girard-Guyonvarc’h, Mathilde Harel, Cem Gabay

**Affiliations:** 1 Division of Rheumatology, Department of Medicine, University Hospital of Geneva, 1206 Geneva, Switzerland; mathilde.harel@unige.ch (M.H.); cem.gabay@unige.ch (C.G.); 2 Department of Pathology and Immunology, School of Medicine, University of Geneva, 1206 Geneva, Switzerland

**Keywords:** interleukin 18, interleukin 18-binding protein, adult-onset Still’s disease, systemic juvenile idiopathic arthritis, macrophage activation syndrome

## Abstract

Interleukin 18 (IL-18) is a pro-inflammatory cytokine of the IL-1 family, whose activity is tightly controlled at the level of production, as well as signalization. Notably, it is buffered by its natural inhibitor, IL-18 binding protein (IL-18BP), which is massively present in circulation in normal and in most pathological conditions, thus preventing harmful pro-inflammatory systemic effects of IL-18. IL-18 has long been considered to be involved in the pathophysiology of various inflammatory diseases. However, a first clinical trial using recombinant IL-18BP for the treatment of rheumatoid arthritis and psoriasis gave disappointing results. Direct measurements of unbound, bioactive, free form of circulating IL-18 demonstrated that IL-18 was more specifically involved in adult-onset Still’s disease (AOSD) and systemic juvenile idiopathic arthritis (sJIA) but also in their most severe complication, macrophage activation syndrome (MAS). More importantly, administration of recombinant IL-18BP to patients with AOSD, and sJIA with MAS, showed promising results. This review summarizes available data regarding IL-18 and IL-18BP in AOSD and sJIA in mouse models and humans and shows the importance of IL-18/IL-18BP imbalance in these conditions, leading to the conclusion that IL-18, particularly free IL-18, may be a useful biomarker in these diseases and an interesting therapeutic target.

## 1. Introduction

Among other biological markers, interleukin 18 (IL-18) appears as a reasonable candidate for a better understanding of adult-onset Still’s disease (AOSD) and systemic juvenile idiopathic arthritis (sJIA) pathophysiology, evaluation of disease activity, and therapeutic approaches. In this review, we will make a brief presentation of IL-18 and IL-18 binding protein (IL-18BP) biology, summarize the fundamental and translational work that has been conducted in the last two decades in this topic, and, finally, state how IL-18 and IL-18BP could be used in clinical practice, for the management of AOSD and sJIA. 

## 2. Interleukin 18, Interleukin 18-Binding Protein, and the Interleukin 18/Interleukin 18-Binding Protein Balance

### 2.1. Interleukin-18: Characterization, Structure, Maturation

IL-18 is a pro-inflammatory cytokine that was first described as an IFN-γ-inducing factor [1]. It is constitutively expressed by various cell types, including monocytes/macrophages, dendritic cells, and epithelial cells [2]. It was suggested to be part of the IL-1 family of cytokines because of its β-pleated structure, which was shown to be similar to the one of IL-1β [3]. Likewise, IL-18 is translated as an immature protein containing an N-terminus pro-domain that must be cleaved for full activity. Among other proteases, caspase-1, the cytosolic enzymatic effector of the inflammasome, has been proposed as the main effector of IL-18 maturation, especially in immune cells [2,4]. As for IL-1β, IL-18 is released in the extra-cellular milieu in its bioactive form through pores formed by oligomerization of Gasdermin D, itself cleaved by caspase-1 [5].

### 2.2. IL-18 Receptor, Signaling Pathways, and IL-18 Functions

**Figure 1:**
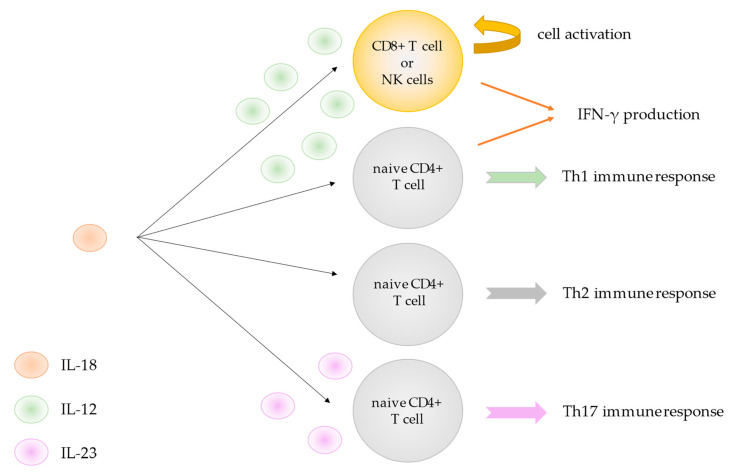
IL-18 functions depending on cytokine environment. IL: Interleukin; CD: cluster of differentiation; NK: natural killer; IFN: interferon. Th: T helper.

The IL-18 receptor (IL-18R) is a heterodimer composed of one binding chain, IL-18Rα, and one signaling chain, IL-18Rβ. IL-18Rα (also known as IL-1R-related protein) is a receptor of the IL-1 family, which binds to IL-18 with low affinity [6]. IL-18Rβ is the co-receptor of the IL-18R and is essential for IL-18 signaling [6]. Both subunits α and β have been proposed to be co-expressed by T cells, dendritic cells, and NK cells, more generally, by competent IFN-γ-producing cells [7]. With IL-18, they form a high-affinity complex that transduces intracellular pro-inflammatory signals. Similar to receptors of the IL-1R family and Toll-like receptors (TLRs), the intracellular region of IL-18Rβ contains a Toll/IL-1 receptor (TIR) domain that, upon IL-18/IL-18R complex formation, binds to the adaptor molecule myeloid differentiation factor (MyD)88 [8] to stimulate an intracellular signaling cascade. Downstream factors include IRAKs, TRAF6, and finally NF-κB [9]. IL-18 mainly targets CD8+ T-cells and NK cells, in collaboration with IL-12, to stimulate IFN-γ production [10] and induce their activation [11,12]. IL-18 also triggers naïve CD4+ T cells differentiation into Th1 cells and IFN-γ release. On the other hand, in the absence of IL-12, IL-18 was shown to promote Th2 rather than Th1 immune responses and, in combination with IL-23, to promote the Th17 pathway (Figure 1) [2,7,13]. Additional functions of IL-18 have been reported, including neutrophil activation and induction of nitric oxide synthesis and VCAM-1 expression [14,15,16]. 

### 2.3. IL-18BP and Interaction with IL-18

IL-18 bioactivity is not only regulated through caspase-1-dependent processing but also by the inhibitory effect of IL-18BP. IL-18BP was first identified in human urine when searching for an IL-18 soluble receptor [17]. It naturally binds mature IL-18 to form high-affinity complexes [18,19]. IL-18BP prevents IL-18 from binding to IL-18R and, thus, IL-18-induced IFN-γ and other pro-inflammatory cytokines production [17,20]. IL-18BP has been shown to be expressed by various immune and non-immune cells in vitro and ex vivo, but the relevant cellular sources of IL-18BP are still unclear in vivo [17,20]. It was found to circulate in a 20-fold molar excess compared to IL-18 in healthy individuals [21]. Consequently, most of circulating IL-18 is bound to IL-18BP and thus inactive. IL-18BP is further upregulated by IFN-γ, which triggers a negative feedback loop in the IL18/Il-18BP balance [22,23]. 

### 2.4. IL-18 in Inflammatory Diseases: The Wrong Track

IL-18 has been considered to play a pathological role in many diseases, according to the data derived from various animal models [24,25,26,27,28]. Furthermore, high circulating levels of IL-18 have been measured in a large number of inflammatory diseases [21,29,30,31,32,33,34], and IL-18 has been shown to be overexpressed in affected organs, such as the intestinal mucosa in Crohn’s disease, the synovial tissue in rheumatoid arthritis, and the skin lesions in psoriasis [35,36,37]. Nonetheless, two clinical trials using recombinant IL-18BP (tadekinig alfa) failed to demonstrate any efficacy of IL-18 blockade in rheumatoid arthritis and psoriasis [38]. Indeed, it appears that, more than the total IL-18 levels, the amounts of free, IL-18BP-unbound, bioactive IL-18 are critical to consider when trying to decipher the role of IL-18 in the pathophysiology of one disease.

## 3. IL-18 and IL-18BP in AOSD and sJIA

AOSD was the first disease where free IL-18 circulating levels were proven to be significantly elevated, thanks to a specifically designed ELISA [19]. Indeed, until then, in some studies, free IL-18 concentrations had been indirectly calculated using the law of mass action, based on the IL-18BP and IL-18 levels, a 1:1 stoichiometry, and a dissociation constant (Kd) of 400 pM [21,29]. Through BIAcore analyses, we showed that the binding affinity of IL-18BP to IL-18 was even higher than previously described [19]. Thus, preceding free IL-18 calculations may have been overestimated.

### 3.1. IL-18 and IL-18BP in Mouse Models of sJIA and Macrophage Activation Syndrome

In opposition to other inflammatory diseases, such as Crohn’s disease or rheumatoid arthritis, data regarding IL-18 and IL-18BP in animal models are scarce. Avau et al. developed a model of systemic inflammatory syndrome compiling most manifestations of sJIA, including arthritis and macrophage activation syndrome (MAS), a well-known complication of sJIA and AOSD. This disease model is induced by injecting complete Freund’s adjuvant in IFN-γ-KO mice. The authors did not find elevated levels of circulating IL-18, only increased IL-18 mRNA expression in lymph node cells (but not in blood cells), as compared to non-injected-IFN-γ-KO mice [39]. Kawane et al. found high serum levels of IL-18 in DNase II-null mice, a mouse model of MAS also displaying arthritis, but demonstrated that the development of arthritis in these mice was IL-18-independent [40]. Conversely, mice repeatedly subjected to TLR9 stimulation develop MAS in an IFN-γ-dependent and IL-18 manner [41]. Indeed, we showed that IL-18BP-deficient mice displayed a more severe phenotype than wild-type (WT) animals and that this was associated with the detection of circulating free IL-18 in IL-18BP-deficient but not in WT mice. In the same MAS model, we demonstrated that depletion of IL-18BP expression in either the radiosensitive or radioresistant compartment did not change the circulating levels of IL-18BP, suggesting that circulating IL-18BP levels are tightly regulated even when some important IL-18BP cellular sources are missing [23]. IL-18 blockade prevented the development of MAS in IL-18BP-deficient mice, the whole indicating that excessive IL-18 signaling is associated with severe manifestations of MAS and that endogenous IL-18BP plays a critical role in regulating IL-18–induced systemic responses [42].

### 3.2. IL-18 and IL-18BP in AOSD and sJIA in Human

For years, it has been reported that IL-18 circulating levels were significantly elevated in AOSD as compared to healthy subjects, other rheumatic diseases, or infections [43,44,45,46]. Similar findings were published in sJIA in comparison to healthy children, non-systemic juvenile idiopathic arthritis, or Kawaski disease [47,48,49,50]. In addition, IL-18 concentrations have been shown to be higher during the active phase of AOSD and sJIA than during disease remission [44,45,46,48,49]. Furthermore, IL-18 levels correlated with AOSD and sJIA disease activity markers, including ferritinemia and CRP [44,45,46,47]. Interestingly, extremely high IL-18 levels have been reported in sJIA patients with a history of MAS [51]. Shimizu et al. also demonstrated that sJIA patients who would later develop MAS had significantly higher IL-18 serum levels than patients who would not [52]. Moreover, higher IL-18 concentrations have been measured in AOSD and sJIA patients with MAS than patients with an active disease without MAS [53,54]. In addition, IL-18 expression was increased in different tissues of AOSD and sJIA patients, including skin, joints, liver, and lymph nodes [50,55,56,57]. Enhanced expression of IL-18 was also described in the bone marrow of a young patient with sJIA, who died with MAS [58].

Of note, polymorphisms in the promoter region of the IL18 gene have been associated with a risk of developing AOSD and sJIA. Diplotype S01/S01 was a major risk factor for susceptibility to AOSD but not to sJIA [59,60]. Nonetheless, sJIA patients carrying S01/S01 showed the highest IL-18 serum levels.

Remarkably, serum levels of IL-18BP were also significantly elevated in patients with active AOSD and sJIA as compared to healthy controls [19,46,48,60]. But no significant difference could be seen between active and inactive AOSD [19,61]. The IL-18/IL-18BP ratio was found to be higher in active sJIA than inactive sJIA patients and healthy controls [48]. More importantly, calculated free IL-18 levels were increased in AOSD and sJIA in comparison to controls, were higher during active than inactive disease, and correlated with markers of disease activity in AOSD [46,61].

**Figure 2:**
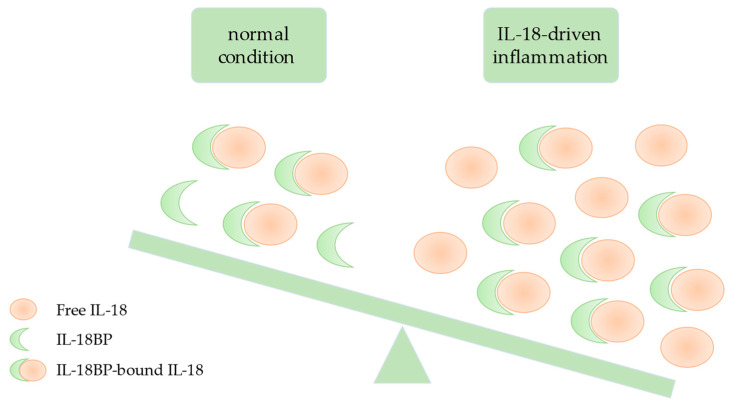
Schematic representation of IL-18/IL-18BP imbalance in AOSD and sJIA. In healthy individuals and most inflammatory diseases, elevated concentrations of IL-18BP and its high affinity for IL-18 causes basically all IL-18 to be buffered by IL-18BP, thus inactive (since unable to bind to IL-18 receptor) (left part of the figure). During active AOSD and sJIA, despite increased circulating levels of IL-18BP, its buffering capacities are overwhelmed by more elevated levels of IL-18 (right part of the figure). Some circulating IL-18 remains unbound to IL-18BP, meaning “free” and biologically active.

By directly measuring IL-18BP-unbound, bioactive, free IL-18, we clearly demonstrated the specific imbalance of IL-18/IL-18BP in AOSD (Figure 2). Indeed, serum levels of free IL-18 were significantly higher in AOSD than in healthy subjects as well as in patients with active rheumatoid arthritis, systemic lupus erythematosus, ankylosing spondylitis, and psoriatic arthritis. Furthermore, we showed that free IL-18 serum levels were higher during active than inactive AOSD and correlated with CRP, ferritinemia, blood leucocyte count, and alanine amino transferase levels [19]. We also found extremely high free IL-18 concentrations in AOSD and sJIA patients with MAS [54,62]. In addition, we reported the case of an sJIA patient with MAS who responded favorably to IL-18 inhibition using tadekinig alfa (human recombinant IL-18BP) [62]. In AOSD, the role of free bioactive IL-18 was further confirmed by a first phase II clinical trial in patients with refractory AOSD who were treated with tadekinig alfa. Half of the patients achieved a response after 3 weeks, as defined by normalization of body temperature and decrease in CRP (by 50% of the baseline levels or <5 mg/L) [63]. We also reported the case of one patient with AOSD, treated with tadekinig alfa, whose high serum levels of free IL-18 became undetectable 2 h after the first subcutaneous administration of IL-18BP, remained low while in disease remission under therapy, and then increased when tadekinig alfa was discontinued, in parallel to AOSD relapse [64].

## 4. Conclusions

More than other inflammatory conditions, AOSD, sJIA, and their dreadful complication MAS are thought to be IL-18-driven. Indeed, it appears that in these diseases, the buffering capacities of IL-18BP are overwhelmed by markedly elevated IL-18 concentrations. A failure of NK cells to respond to IL-18 and produce IFN-γ, leading to decreased IL-18BP levels, has been hypothesized to explain this IL-18/IL-18BP imbalance [65]. More than total IL-18 levels, free IL-18 concentrations appear as a useful marker of AOSD, sJIA, and MAS, that could help physicians to establish the diagnosis of these challenging conditions. Furthermore, IL-18 may be an interesting therapeutic target in AOSD and sJIA, with or without MAS. A phase III clinical trial using tadekinig alfa in active AOSD is about to begin.

## Data Availability

Not applicable.
